# Quality Attributes and Sensory Acceptance of Different Botanical Coffee Co-Products

**DOI:** 10.3390/foods12142675

**Published:** 2023-07-11

**Authors:** Katarína Poláková, Alica Bobková, Alžbeta Demianová, Marek Bobko, Judita Lidiková, Lukáš Jurčaga, Ľubomír Belej, Andrea Mesárošová, Melina Korčok, Tomáš Tóth

**Affiliations:** Institute of Food Sciences, The Faculty of Biotechnology and Foods Sciences, The Slovak University of Agriculture in Nitra, Tr. A. Hlinku 2, 94976 Nitra, Slovakia; xpolakovak1@uniag.sk (K.P.); xdemianova@uniag.sk (A.D.); marek.bobko@uniag.sk (M.B.); judita.lidikova@uniag.sk (J.L.); xjurcaga@uniag.sk (L.J.); lubomir.belej@uniag.sk (Ľ.B.); xmesarosova@uniag.sk (A.M.); xkorcok@uniag.sk (M.K.); tomas.toth@uniag.sk (T.T.)

**Keywords:** cascara (coffee husks), silverskin (thin layer), *Coffea arabica*, *Coffea canephora*, quality, bioactive compounds, physical–chemical parameters, sensory analyses

## Abstract

Coffee processing is a major contributor to the creation of food and product waste. Using coffee co-products can play an essential role in addressing environmental problems and issues with nutritionally unbalanced foods, population growth, and food-related diseases. This research aimed to determine the quality and sensory parameters (aw, pH, dry matter, TAC, TPC, fat, fatty acids profile, fiber, caffeine, chlorogenic acids, color, and sensory analysis) of different botanical origins of cascara (coffee husks) and silverskin (thin layer). The results of this study show that silverskin and cascara are a good source of TAC (1S 58.17 ± 1.28%, 2S 46.65 ± 1.20%, 1C 36.54 ± 1.84%, 2C 41.12 ± 2.11%). Cascara showed the presence of polyphenols (2C 49.135 g GAE·kg^−1^). Coffee co-products are good sources of fiber. Silverskin had higher values of caffeine than cascara. Palmitic, stearic, oleic, linoleic, and arachidic acids were the most represented acids in the samples. Given the obtained results, cascara can be considered “low-fat” (1C 4.240 g·kg^−1^ and 2C 5.4 g·kg^−1^). Based on the sensory evaluation, no sample reached the acceptable index value of 70%. Understanding the link between the character, identification properties, and composition of coffee co-products of different botanical origins can enable their application in the food industry.

## 1. Introduction

The consumption of coffee has significantly increased over the last decade, and the coffee industry is generating increasing amounts of food waste [1]. The most crucial co-product of the early stages of post-harvest coffee processing is cascara (coffee husks). Silverskin (thin layer) is another important co-product created using the coffee bean roasting process. Both co-products are currently considered waste [2,3]. The disposal of co-products created during the technological processing of coffee cherries is currently an environmental problem [4]. Therefore, the coffee processing industry focuses on valorizing co-products created during post-harvest operations that are usually considered food waste [5]. There are two main methods of coffee processing: dry and wet [6]. Wet processing means that after harvesting coffee cherries, the skin and pulp are fermented or enzymatically split to yield the seeds. During the dry processing method, coffee cherries are sun-dried, and the outer layers of the coffee cherry are mechanically separated from the green beans [7]. Knowledge of the anatomical parts of the cherry is vital for accurately characterizing the individual parts of the coffee cherry and comparing the content compositions of these parts [8]. The definition and composition of cascara depend on the type of processing employed: the wet or dry method [9]. Esquivel and Jiménez [10] described two definitions of cascara based on the processing method. If it is obtained through wet processing, the cascara is formed by the coffee cherry’s smooth outer layer, which is green in unripe fruits, red in ripe fruits (exocarp 12%), and the pulp (outer mesocarp 29%). Following the formation of a highly hydrated mucilage layer (pectin layer 8%) and a thin yellowish endocarp called parchment (6.1%), the cascara coffee co-product is formed if obtained during dry processing. The roasting process creates another co-product that is called silverskin. Silverskin remains attached to the green coffee grains after processing and falls off due to the high temperatures during roasting [11]. Cascara accounts for up to 45% of the coffee cherry [9,10], while silverskin only makes up approximately 1% [12].

In recent years, we have observed increased demand for functional food with attractive nutritional compositions. Especially sought after are food products with high antioxidant activity, high dietary fiber, and low-fat contents [3]. Research shows that coffee cherries and their coffee co-products contain valuable nutrients. Carbohydrates, dietary fiber, protein, fat, minerals, and vitamins comprise most coffee co-products nutritional composition. These properties make cascara and silverskin potential sources of nutrients that can be used in novel food development [13,14].

As mentioned above, previous studies on coffee co-products indicate that coffee co-products are rich in valuable bioactive compounds. Coffee co-products could be used to develop and create a novel food product. Another reason to use co-products is to minimize waste from the coffee industry. Based on worldwide population growth and the higher incidence of people with intolerance and allergies to food, there is a need to focus on creating innovative, nutritionally attractive food and utilizing new plant-based material in novel food. Our work aimed to perform a pilot study of coffee co-products that differ in their botanical origins in order to discover novel information regarding their properties, quality, and possible consumer acceptance when used in novel food products.

## 2. Material and Methods

For the purpose of this research, four samples of coffee co-products of different botanical origins were used: cascara, n = 2 (*Coffea arabica*, *Coffea canephora*) and silverskin n = 2 (*Coffea arabica*, *Coffea canephora*). These samples were provided by the local company focused on importing and distributing coffee, Caffé Oro Ltd. (Zvolen, Slovakia). Detailed information about the analyzed samples is listed in Table 1. Pictures of the analyzed samples are included in the Supplementary Material (Figures S1 and S2).

### 2.1. Roasting Process

Silverskin analyzed in the study was obtained during the roasting of Coffea arabica green coffee beans from Panama and *Coffea canephora* (India). The roasting process was performed using barrel roaster technology with a gas heating system using a Toper TKSMX 10 machine (Toper, Izmir, Turkey). The beans were roasted to a medium roast under the following conditions: The initial temperature was set to 180 °C for 3 min. After the initial phase temperature was raised to 205 °C, and after 4 min, the first crack occurred, and the temperature reached 215 °C.

### 2.2. Grinding and Extract Preparation

All samples were ground at 10,000 rpm for 60 s using a Grindo-mix GM 200 (Retsch, Haan, Germany). The homogenized samples were then sieved through a sieve with a mesh diameter of 1 mm (standard laboratory test sieve, unbranded). The coffee co-products were extracted using deionized water at a temperature of 95 °C. Samples were extracted for 5 min with occasional stirring. Extraction was followed by filtration through Sartorius filter paper (Sartorius Lab Instruments GmbH & Co. KG, Gottingham, Germany, WHA1001125 Whatman qualitative filter paper, Grade 1, circles, diam. 125 mm), thus obtaining final solutions, which were used for chemical and sensory analyses.

### 2.3. Determination of Water Activity (a_w_)

We used the Water Activity Meter Fast-Lab (Germany) to determine the water activity a_w_ of analyzed samples. The measurement was performed in triplicate individually for each sample.

### 2.4. Determination of pH

After extraction, we determined the pH value in the samples at 20 °C using a pH 70 portable pH meter (XS Instruments, portable pH meter pH7 + DHS Kit − Non-DHS electrode, Italy, Modena). The pH was measured in triplicate on each sample. Before analysis, the pH meter was calibrated using Hamilton Buffer Solutions (Hamilton Bonaduz AG, Bonaduz, Switzerland) with a pH of 4.01, 7.0, and 10.01.

### 2.5. Determination of Dry Matter

The dry matter content of the cascara and silverskin powder samples was determined using KERNDAB 100-3 moisture analyzer (KERN & SOHN GmbH, Balingen, Germany). The drying program was set at 110 °C. The dry matter was expressed in %.

### 2.6. Determination of Total Antioxidant Capacity (TAC)

The DPPH (2,2-diphenyl-1-picrylhydrazyl) radical scavenging activity assay methodology was adapted from Brand-Williams et al. [15]. The first step in this method was to weigh 0.025 g of DPPH radical and dissolve it in ethanol (Centralchem, Bratislava, Slovakia, 96%). Subsequently, the volumetric flask with the stock solution was filled up to 100 mL. The stock solution was diluted with ethanol (1:9). A total of 3.9 mL of diluted DPPH solution was transferred into the glass cuvettes, and the initial DPPH absorbance (*A*_0_) was measured at a wavelength of 515.6 nm. Next, 100 μL of sample extract was pipetted into a cuvette, and the mixture was stirred with a glass rod. The absorbance (*A_t_*) was measured after 10 min at 515.6 nm (T80 UV/VIS Spectrometer; PG Instruments, Ltd.; Lutterworth, UK). We determined the antioxidant activity of the extracts as a percentage of the inhibition of DPPH radicals. The scavenging capacity was calculated using the following equation:

$$\%inhibition\;DPPH=\frac{\left(A_{0}-A_{s})-(A_{t}-A_{s}\right)}{\begin{array}{c} \left(A_{0}-A_{s}\right)\times 100 \end{array}}$$

where

- *A*_0_ is the initial absorbance of the DPPH solution;
- *A_s_* is the absorbance of ethanol (blank);
- *A_t_* is the absorbance after 10 min.

### 2.7. Determination of Total Polyphenols Content (TPC)

A methodology using the Folin—Ciocalteu reagent for the TPC determination was adopted from the work of Fu et al. [16]. 

The content of TPC was expressed as grams of gallic acid equivalents (GAE) per kilogram of dry matter. For this analysis, a double-beam UV-VIS spectrophotometer (T80 UV/VIS Spectrometer; PG Instruments, Ltd.; Lutterworth, UK) equipped with an eight-place cuvette holder was used. The glass cuvettes were type S/G/10 (Exacta+Optech GmbH, Munich, Germany).

To prepare a stock solution, 100 mg of gallic acid was weighed (Sartorius Lab Instruments GmbH & Co. KG, Göttingen, Germany), diluted with demineralized water, and filled up to 100 mL in a volumetric flask. Exactly 1 mL of the stock solution was diluted with distilled water up to 200 mL volume. The calibration curve was prepared in between 5 and 200 mg·L^−1^ of gallic acid. The blank contained a Folin—Ciocalteu reagent and distilled water without the standard or extract. The correlation coefficient of the calibration curve reached *R*_2_ = 0.996.

Before the measurement, samples were prepared as follows: 50 μL of extracts were pipetted into 50 mL volumetric flasks. The Folin–Ciocalteu reagent was diluted with distilled water (1:2 *v/v*), and 2.5 mL of this solution was added to a flask with the extract. Next, 5 mL of Na_2_CO_3_ (20% water solution) was added. Flasks were filled with distilled water up to 50 mL and left for two hours at room temperature to develop the blue-colored complex. Samples absorbance was measured at the wavelength set to 765 nm.

### 2.8. Determination of Fat and Fatty Acids Profile

Fat was extracted from the lyophilized samples using Soxhlet extraction with petroleum ether. The methodology for the Soxhlet extraction with petroleum was adapted from Luque de Castro et al. [17] and modified by ISO 12966-2:2017: preparation of methyl esters of fatty acids, animal and vegetable fats, and oils [18]. The fat content was expressed as g·kg^−1^ of dry matter and fatty acids methyl ester profile in %. 

The chemical reagents used for fat and fatty acid determination were: petroleum ether 40–65 °C (Sigma–Aldrich, Steinheim, Germany), methanol for chromatography (Sigma–Aldrich, Steinheim, Germany), NaHSO_4_ H_2_O (Centralchem, Bratislava, Slovakia), Na_2_SO_4_ (Centralchem, Bratislava, Slovakia), KOH (Centralchem, Bratislava, Slovakia), HCl 33–36% (Centralchem, Bratislava, Slovakia), filter medium Hyflo Super Cel (diatomaceous earth) (Sigma–Aldrich, Steinheim, Germany), anhydrous N-hexane for chromatography (Sigma–Aldrich, Steinheim Germany), 42 component standard FAME mix 10 mg·mL^−1^ in methylene chloride containing C4-C24 FAME (2–4% relative concentration), manufacturer Supelco, catalog number 47885-U chromatography (Sigma–Aldrich, Steinheim, Germany). Apparatus: vacuum oven (Fisher Scientific, Waltham, MA, USA) temperature adjustable (100 ± 3 °C). Tecator Soxtec System HT 1043 Extraction unit (Gemini, Apeldoom, the Netherlands) apparatus at a flow rate of 10 cycles/h or 10 mL·min^−1^.

#### Preparation of Fatty Acid Methyl Esters (FAME)

The amount of each sample was 10 g. We used the obtained fat to prepare methyl esters (FAME): 200 mg of fat was transferred into a test tube using a micropipette. The pipetted volume on the micropipette corresponded exactly to the weight. 

We added 5 mL of n-hexane to the fat sample and dissolved the fat by stirring. We added 1 mL of 2 M solution KOH in methanol. We shook the tube contents vigorously and placed the tubes in a water bath set at 60 °C for 30 s. The sample was then allowed to rest for 1 min, then 2 mL of 1 M HCl was added, and the tube contents were mixed gently. After equilibrium was reached and the layers were separated, the upper layer containing the methyl esters was carefully filtered through anhydrous Na_2_SO_4_ and used for the analysis. Before GC-FID analysis, we diluted the acid-containing methyl ester solutions into the vial in the ratio of 50 uL of FAME solution: 950 μL n-hexane. 

The GC-FID was performed using Agilent 6890 GC with FID (flame ionization detector), and an analytical column: 60 m × 250 μm × 0.15 μm DB-23 (Agilent 122–2361) was used. Experimental conditions were set as follows: injector temperature: 250 °C; injected volume: 1 μL; dividing ratio: 1/10; carrier gas: helium; head pressure: 238.96 kPa (2.225 mL·min^−1^); temperature program: 50 °C for 1 min, 25 °C min^−1^ until the system reached 175 °C, then 2 °C min^−1^ to 230 °C; detector temperature: 280 °C; gas detector: hydrogen: 35 mL·min^−1^, air: 350 mL·min^−1^, nitrogen; make-up gas: 30 mL·min^−1^. 

The chromatograms of our analyzed samples were compared with each other and with the chromatogram of the standard. The quality indicator was the elution time of the separated analytes. The quantity indicator is the area under the peak of the analyte of interest. The internal normalization method was used for quantitative evaluation. Provided that all components of the sample were recorded on the chromatogram, representing a total peak area of 100%. The areas under the individual peaks, i.e., the individual fatty acid methyl esters, represented the weight percentage of a given fatty acid of the total fatty acid content present in the sample [19].

### 2.9. Determination of Fiber

The total dietary fiber content was determined using an enzymatic gravimetric assay based on the AACC [20]. To perform the analysis, 1 g of each sample was weighed and mixed with 50 mL of a phosphate buffer solution (pH 6) and 50 µL of an α-amylase solution. The mixture was placed in a boiling water bath for 15 min (with occasional stirring). After cooling to room temperature, the pH was adjusted to 7.5 with 10 mL NaOH prepared, and 100 µL of the protease solution was added. The mixture was incubated for 30 min at 60 °C with constant stirring. After cooling, the pH was adjusted to 4.5 with 10 mL of HCl, and 200 µL of amyloglucosidase solution was added. The mixture was again incubated for 30 min at 60 °C with constant stirring. Subsequently, 280 mL of isopropanol (preheated to 60 °C) was added, and the mixture was left at room temperature for 60 min (a precipitation reaction was taking place). After filtration, the precipitate was collected and washed three times with 20 mL of 78% ethanol, twice with 10 mL of 95% ethanol, and twice with 10 mL of acetone. The filter paper with the residue was left at 105 °C for 12 h, and then the weight of the residue was determined. The protein content was determined on one residue sample. The ash content was determined in the second residue sample (5 h at 525 °C in a muffle furnace). The amount of fiber in % was calculated as follows:

$$\mathrm{Fiber}\;\%=\frac{\frac{R_{1}+R_{2}}{2}-p-A-B}{\frac{m_{1}+m_{2}}{2}}\times 100$$

where

- *R*_1_ = weight of residue 1;
- *R*_2_ = weight of residue 2;
- *m*_l_ = weight of sample 1;
- *m*_2_ = weight of sample 2;
- *A* = ash content from the *R*_1_ residue;
- *p* = protein content of the *R*_2_ residue;
- *B* = blank.

The results were expressed as weight percent (%).

### 2.10. Determination of Chlorogenic Acids and Caffeine by HPLC-DAD

The methodology for the determination of caffeine and chlorogenic acids by HPLC-DAD (High-Performance Liquid Chromatography with Diode-Array Detection) was adapted from Bobková et al. [21]. Analyses were performed using the HPLC Agilent Infinity 1260 (Agilent Technologies GmbH, Waldbronn, Germany) equipped with a DAD detector (1260 DAD VL+). The separation was performed using a LiChroCART 250-4 Purospher STAR, with an RP-18 end-capped column (250 mm × 4 mm × 5 µm; Merck KGaA, Darmstadt, Germany). Methanol (A) and 0.1% solution of formic acid in ddH_2_0 (*v/v*) (B) were used as a mobile phase. The separation was performed at gradient elution (0–2 min: 20% A + 80% B); in the 2–15 min period, the ratio of mobile phases was gradually changed to a final value of 40% A + 60% B, and the post time and equilibration were changed back (20% A + 80% B: 3 min). The flow rate was 1 mL min^−1^, and the injection volume was 3 µL. The temperature was set at 40 °C. The detection wavelengths were set at 240 and 280 nm. Obtained data were processed using the Agilent OpenLab ChemStation program. The following reagents were used as standards: caffeine, chlorogenic acid, neochlorogenic acid, cryptochlorogenic acid, dicaffeoylquinic acid, 3,5-dicaffeoylquinic acid, and 4,5-dicaffeoylquinic acid.

### 2.11. Color Determination

The present study used the colorimeter (CR 400, Minolta, Tokyo, Japan) to determine the sample color. The white plate calibration was performed according to the colorimeter manual. A compact layer of homogenized samples in powder form was placed in a Petri dish. The color was then measured randomly from the central part of the Petri dish three times. Using the spectrophotometer, three color parameters were measured: lightness (L*), redness (a*), and yellowness (b*). Another two parameters were calculated: chroma (C* = square root of (a*² + b*²)) and hue angle (h° = tan inverse (b*/a*)). In our measurement, we used option SCI (Specular Component Included) [22].

### 2.12. Sensory Analysis

Sensory analysis is one of the most well-known tools for assessing consumer acceptability of various products. During the sensory analysis, prepared extracts were evaluated using a nine-point hedonic scale focusing on the following parameters: appearance, bitterness, acidity, aroma, taste, coffee aftertaste, and tea aftertaste. Tea aftertaste was selected given that coffee co-products (cascara and silverskin) originate from coffee, but they are sometimes described as tea. Attributes were selected based on the studies of Barahona et al., Pereira et al., DeSousa et al., Riandani et al., Meilgaard et al., and DePaula et al. [23,24,25,26,27,28]. The score on this scale ranges from 1 to 9 (1—very bad, 9—very good). The acceptability index (AI) was calculated as part of the evaluation. An AI equal to or greater than 70% is considered satisfactory [27]. The index was calculated using the following equation:

$$\mathrm{AI}=\frac{X\times 100}{N}$$

where

- *X* = average score awarded by evaluators;
- *N* = highest score awarded by evaluators.

A total of 12 semi-trained evaluators recruited from the Slovak University of Agriculture in Nitra participated in this pilot study. The panel had previous experience with sensory evaluation and using hedonic scales during their education, as well as with coffee evaluation. Of them, 42% of evaluators were male, and 58% were female. Additionally, 92% of evaluators were in the age range of 18–30, and 8% were in the age range of 45–60. The evaluators reflect on their habits related to the consumption of coffee and other caffeinated beverages in the questionnaire. A total of 92% of the evaluators reported consuming coffee; the same number said they drink tea. Likewise, 1% of panelists are smokers, and 42% consume energy drinks regularly.

### 2.13. Statistical Analysis

Obtained data were analyzed using the XLSTAT [29]. ANOVA analysis with Duncan and REGWQ tests were used. For all the tests, the significance α was set to 0.05. Measured data were presented as mean values of three replications ± standard deviations for all determined parameters. Linear Discriminant Analysis (LDA) was used to describe any possible significant differences between the analyzed samples. In statistical analysis, we aimed to find a low-dimensional representation that maximizes class separability; therefore, we used LDA. This statistical method is commonly used for analysis, classification, and reduction. LDA reduces the dimensionality of the data by maximizing class separability. This technique is a supervised learning algorithm. This means LDA finds directions of maximum class separability [30,31]. Pearson’s correlation was used for the correlation between chemical parameters and sensory attributes.

## 3. Results and Discussion 

Results provide an overview and discussion of each parameter. In our study, we determined various physico-chemical parameters of coffee co-products. Information in Table 2 describes different measured parameters, which will be mentioned and discussed within their corresponding sections (3.1 water activity, 3.2 pH, 3.3 dry matter, 3.4 TAC and TPC, 3.6 fiber content, 3.7 chlorogenic acids, and caffeine).

### 3.1. Water Activity (a_w_)

Water activity in food is defined as the energy state of water. This parameter is one of the factors influencing the chemical and biochemical reactions and growth or presence of microorganisms in food and raw materials. It is one of the parameters used to define and potentially estimate the stability and safety of food and raw materials regarding the growth and reproduction of microorganisms and changes in their chemical and physical properties. Water activity can affect the quality of raw materials, final food quality, safety, stability, and technological properties [32,33].

Water activity values were significantly lower in silverskin (1S 0.18 and 2S 0.28) compared to cascara (1C 0.64 and 2C 0.39). These results also provide important insights that there were differences in water activity values in cascara and silverskin samples depending on botanical origin. Silverskin *Coffea arabica* (1S) had a lower water activity than silverskin *Coffea canephora* (2S). The opposite was observed between cascara samples. Cascara *Coffea arabica* (1C) had higher water activity than cascara *Coffea canephora* (2C). According to these data, we can assume that botanical origin can affect water activity in coffee co-products.

### 3.2. pH

The measured pH values of coffee co-products of different botanical origins (*Coffea arabica* and *canephora*) shows that *Coffea canephora* co-products (2S, pH 5.90; 2C, pH 5.97) had a higher value of pH than *Coffea arabica* co-products samples coffee co-products (1S, pH 4.62; 1C, pH 4.22). Differences in pH values between cascara and silverskin can be explained by roasting, which influences the pH value of silverskin [34].

Martuscelli et al. [35] determined the pH value of silverskin from *Coffea arabica* as 5.34 ± 0.02, representing a higher value than our findings (1S, pH 4.62). Another study determined the pH value of silverskin aqueous extract as an average value for both *Coffea arabica* and *Coffea canephora* at 5.00. A possible explanation for this might be the different origins of the analyzed samples in the given study [36].

Murlida et al. [37] reported a pH value of *Coffea arabica* cascara extract of 5.30. Similarly, higher values were determined by Prono-Widayat et al. [38], who reported the range (4.67–5.90) of pH values of cascara *Coffea arabica* depending on the fermentation process, which corresponds with our results.

Maharani et al. (2021) [39] found that different leaching times affect the pH value of cascara extract.

### 3.3. Dry Matter

Considering that silverskin is produced during the roasting process and is derived from coffee beans, its dry matter content is expected to be similar to roasted coffee beans [40]. 

This statement accords with our observations that the dry matter of silverskin from *Coffea arabica* was 92.70%, and 93.91% from *Coffea canephora.* When converting the value of silverskin’s dry matter to the value of the moisture content, silverskin represents a stable raw material [41]. Bertolino et al. [42] reported the dry matter content of *Coffea arabica* silverskin in the range of 93.95–95.73%. Similar values were reported by Gottstein et al. (2021) [33]. They determined the dry matter of the *Coffea arabica* silverskin samples at 93.85 ± 0.12%. These values show similarity with our data. 

The dry matter value of cascara *Coffea arabica* was 85.16%, and *Coffea canephora* was 89.60%. Similar findings of dry matter content in cascara are reported in the technical report on the safety of dried coffee husks (cascara) from *Coffea arabica* L. in Regulation (EU) 2015/2283, namely 83.5–87.9% [43].

Other values of the dry matter content of cascara comparable to our results are reported by Braham and Bressami [44]. They reported a cascara dry matter value in the 87.4–92.1% range.

### 3.4. Total Antioxidant Capacity (TAC) and Total Polyphenols Content (TPC)

One of our goals was to determine the total antioxidant capacity of coffee co-products. Silverskin reached significantly higher TAC values (1S 58.17% and 2S 46.65%) than cascara (1C 36.54% and 2C 41.12%). Maharani et al. [39] reported the TAC value of cascara in the range of 61.01–87.30%, depending on the time and method of extraction. These results differ from our findings. This discrepancy could be attributed to the different geographic origins of samples in the authors’ study, which were from Java. Sholichah et al. [45] reported fermentation as another factor influencing the TAC value of cascara. Very little was found regarding the content of the TAC of silverskin in the scientific literature.

Another parameter determined in coffee co-product samples in our study was total polyphenols content (TPC). The most striking finding was the substantial difference in the TPC between samples of cascara and silverskin due to their botanical origin. Coffee co-products of *Coffea canephora* had a significantly higher TPC (2S 11.15 g GAE·kg^−1^ and 2C 49.14 g GAE·kg^−1^) compared to *Coffea arabica* co-products (1S 1.64 g GAE·kg^−1^ and 1C 2.59 g GAE·kg^−1^). Arpi et al. [46] reported that TPC content varies in different varieties of coffee cherry. Sholichah et al. [45] also observed the effect of fermentation on the TPC content of cascara.

### 3.5. Fat and Fatty Acid

During the analysis, we determined 12 out of 42 individual fatty acids. Of this amount, 5 fatty acids were not specified. Butyric acid, caproic acid, caprylic acid, capric acid, undecanoic acid, lauric acid, trid canoic acid, pentadecanoic acid, cis-10-pentadecenoic acid, palmitoleic acid, heptadecanoic acid, cis-10-heptadecenoic acid, elaidic acid, linolelaidic aciid, γ-linolenic acid, cis-11-eicosenoic acid, cis-11,14-eicosadienoic acid, cis-8,11,14-eicosatrienoic acid, arachidonic acid, cis-11,14,17-eicosatrienoic acid, cis-5,8,11,14,17-eicosapentaenoic acid (EPA), erucic acid, cis-13,16-docosadienoic acid, nervonic acid, cis-4,7,10,13,16,19-docosahexaenoic acid (DHA) are acids that were not identified in analyzed samples. The values of these fatty acids were under the detection limit. Chromatograms of fatty acids measured using GC-FID (Figures S3 and S4) and their profiles are shown in the Supplementary Material (Table S1). 

**Figure 1:**
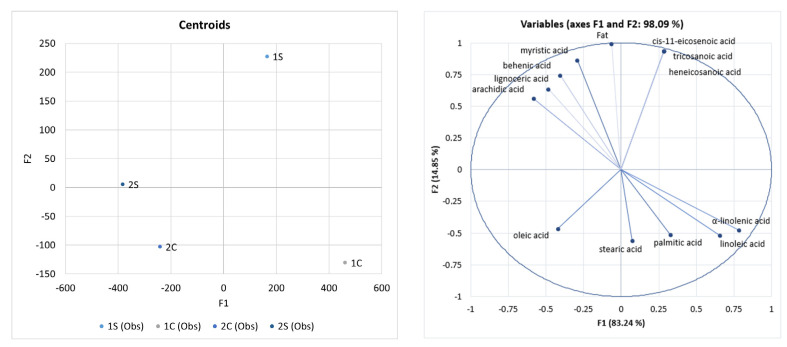
Differentiation of coffee co-products based on FAME using Linear Discriminant Analysis (LDA), fat (g·kg^−1^), and fatty acids (%). Note: 1S—Silverskin (thin layer) *Coffea arabica*; 2S—Silverskin (thin layer) *Coffea canephora*; 1C—Cascara (coffee husks) *Coffea arabica*; 2C—Cascara (coffee husks) *Coffea canephora*.

According to the LDA (Figure 1), silverskin *Coffea arabica* is a good source of cis-11-eicosenoic acid, heneicosanoic acid, and tricosanoic acid. Angeloni et al. [47] identified the following fatty acids in silverskin behenic (11%) and arachidic (9%). Their values are comparable to our data. 

In our study, arachidic acid, lignoceric acid, behenic acid, and myristic acid were typically identified as acids for silverskin *Coffea canephora.* Gottstein et al. [40] identified behenic acid and arachidic acid as the main constituents of silverskin (*Coffea canephora*). This report is consistent with our claims, given that our results showed that silverskin is the source of behenic and arachidic acids.

Our data showed that α-linolenic acid, linoleic acid, palmitic acid, and stearic acid were identified significantly in cascara *Coffea arabica*. Our study showed that cascara *Coffea canephora* was a good oleic acid source. Rios et al. [48] identified arachidic acid, palmitic acid, and stearic acid as the main constituents of cascara. This claim is consistent with our results.

The value of fat content for cascara *Coffea arabica* (4.77 g·kg^−1^), cascara *Coffea canephora* (2.4 g·kg^−1^) was significantly lower than fat in silverskin *Coffea arabica* (25.74 g·kg^−1^), silverskin *Coffea canephora* (13.550 g·kg^−1^). Fat properties are influenced by the composition of fatty acids [49]. Differences in silverskin lipid content can be partially attributed to factors such as different varieties of coffee, the origin of coffee beans, coffee-growing conditions (climate, time of harvest), and processing methods (wet or dry processing and roasting) [40,50,51]. Gottstein et al., Mota et al., and Efthymiopoulos et al. [40,50,51] reported the fat content in silverskin (*Coffee canephora*) reaching 18.2 ± 0.1 g·kg^−1^. Costa et al. [12] reported that silverskin contains 24.2 g·kg^−1^ of fat. We achieved similar results in our research. When comparing the fat content between the group of coffee co-products, we can observe lower fat values in cascara. This claim is consistent with the following findings. Due to the low-fat content of cascara, an “instant cascara” drink was developed with the following nutritional claims: “low fat”, “low sugar,” and “source of potassium and vitamin C” [9].

Identified fatty acids in our analyzed samples were divided into three groups (polyunsaturated fatty acids—PUFA, monounsaturated fatty acids—MUFA, and saturated fatty acids—SFA). These results show in Table 3.

The group of PUFA consists of the following acids: linoleic acid, linoleic acid, γ-linolenic acid, α-linolenic acid, cis-11,14-eicosadienoic acid, cis-8,11,14-eicosatrienoic acid, arachidonic acid, cis-11,14,17-eicosatrienoic acid, cis-5,8,11,14,17-eicosapentaenoic acid (EPA), cis-13,16-docosadienoic acid, cis-4,7,10,13,16 acid, and 19-docosahexaenoic acid (DHA). A group of MUFA consists of myristic acid, cis-10-pentadecenoic acid, palmitoleic acid, cis-10-heptadecenoic acid, elaidic acid, oleic acid, cis-11-eicosenoic acid, erucic acid, and nervonic acid. A group of SFA is represented by: butyric acid, caproic acid, caprylic acid, undecanoic acid, lauric acid, tridecanoic acid, myristic acid, pentadecanoic acid, palmitic acid, heptadecanoic acid, stearic acid, heneicosanoic acid, behenic acid, tricosanoic, lignoceric acid, and arachidic acid.

Efthymiopoulos et al. [51] observed the content of these groups in silverskin (*C. arabica*) in the following order: SFA (55.5–60%) > PUFA (31.5–36.1%) > MUFA (8.3–8.4%). Considering our results, we can confirm this statement. Silverskin had significantly higher values of SFA compared to cascara [12]. Silverskin contained higher levels (1S 12.286 and 2S 6.059) of the Σn6/Σn3 ratio compared to the cascara sample (1C 1.504 and 2C 0.000). Similar values were reported by Rios et al. [48]. The same authors determined Σn6/Σn3 ratio in cascara reaching the value 1,25, which is in accordance with our results (sample 1C). The value of ratio Σn3/Σn6 ratio Σn6/Σn3 in our analyses was in sample 2C under the detection limit.

### 3.6. Fiber Content

The dietary fiber content in silverskin was 64.73% in the 1S sample and 61.06% in the 2S sample. Silverskin *Coffea arabica* shows higher fiber values compared to *Coffea canephora* silverskin. The same was observed by Giordano et al. [52]. The authors also claim that the high fiber level found in the silverskin supports the hypothesis that silverskin has great potential as a functional food ingredient. This opinion is also shared by Gottstein et al. [40].

Santos et al. [53] reported a total fiber content of 60.50% in cascara and 60.00% in silverskin. However, this research does not indicate the variety of analyzed co-products. We obtained comparable results of fiber content in silverskin for both *Coffea arabica* and *Coffea canephora,* as well as for cascara from *Coffea canephora.* Rodriguez et al. [54] reported a similar fiber content in silverskin (56–62%). However, Loorbeer et al. [36] reported a much wider range (34.70–68.50%).

The samples of cascara *Coffea arabica* analyzed in our study contained 37.52% of dietary fiber. Jiamjariyatam et al. [55] reported lower values, 23%, in an unspecified cascara sample. After the different post-harvest processing, Mindarti et al. [56] reported that *Coffea arabica* reached different content of dietary fiber (56.7 ± 0.54% in the dry-processed form, and 63.16% ± 0.56% in the wet form of post-harvest processing).

The cascara *Coffea canephora* (2C) contained 63.75%, and the silverskin *Coffea canephora* (2S) 61.06% of dietary fiber. Iriondo-DeHond et al. [9] studied the silverskin from *Coffea arabica* and *Coffea canephora* separately and reported a total dietary fiber content of 67.70% for silverskin *Coffea arabica* and 69.30% for silverskin *Coffea canephora.* The authors also measured the total fiber content of silverskin obtained from a mixture of *Coffea arabica* and *canephora* at 62.40%. Rios et al. [48] analyzed wet-processed cascara from Colombia (*Coffea arabica,* variety Tabi) and determined that the total dietary fiber content reached 47.44 ± 1.85%. Our results are comparable with the findings of Mindarti et al. [56]. Their study reported a fiber content in wet-processed at 65.98 ± 0.14 and in dry form at 73.32 ± 0.20. Hejna [57] reports a fiber content, for an unspecified type of silverskin, in the range of 56.4–71.90%. Similar data on fiber content are also reported by Cantele et al. [58]; 57.18 ± 4.95% in the case of *Coffea canephora* silverskin. Our data are in line with the findings of the authors mentioned above.

### 3.7. Chlorogenic Acids (CGAs) and Caffeine

Silverskin is created during coffee roasting, meaning it is exposed to high roasting temperatures. Farah and Lima [59] stated that CGAs are thermolabile compounds; thus, a significant amount is degraded during the roasting. Król et al. (2019) [60] also reported a decrease in CGAs concentration due to the roasting. This hypothesis is further supported by Fuller and Rao [61]. Hence, it could be hypothesized that silverskin would have a lower content of chlorogenic acids than cascara, which is not exposed to the roasting process. This statement is in line with our results, given that the concentration of CGAs detected in silverskin (1S 0.38 mg·g^−1^ and 2S 0.50 mg·g^−1^) was significantly lower than in cascara samples (1C 0.60 mg·g^−1^ and 2C 0.52 mg·g^−1^). The measured content of neochlorogenic acid, cryptochlorogenic acid, dicaffeoylquinic acid, and 4,5-dicaffeoylquinic acid showed significant differences (*p* ≤ 0.05) in our samples. 

Furthermore, we observed the absence of 3,5-dicaffeoylquinic acid in samples 1S, 2S, and 1C and the absence of 4,5-dicaffeoylquinic acid in samples 1S and 2C. Mandura et al. (2021) [6] also reported similar phenomena. Moreover, the same authors found that the caffeine content of cascara reached 9.20 ± 0.50 mg·g^−1^ and silverskin 5.30 ± 0.2 mg·g^−1^. Therefore, the authors defined cascara as a better caffeine source than silverskin. These findings contradict our findings. Our work detected significantly higher caffeine content in silverskin compared to cascara, regardless of the botanical origin.

The main factors affecting the caffeine content of cascara include the fermentation process, botanical origin, variety, post-harvest processing, time, and method of extract preparation [6,46,62]. Comparable results of caffeine content in silverskin reaching 6.50 ± 0.60 mg·g^−1^ were published by Cantele et al. [58]. 

### 3.8. Color Determination

The next section of the research was dedicated to instrumental color determination. L* represents the lightness measured as brightness, with 100 and 0 values corresponding to absolute white and black, respectively. The a* represents redness, and b* yellowness [63]. The results of instrumental color determination are shown in Table 4.

Regarding the lightness level in cascara samples, we can observe a higher measured value of L* in 1C *Coffea arabica* (30.07) than in 2C *Coffea canephora* (28.65). The measured redness (a*) and yellowness (b*) were also higher in the 1C *Coffea arabica* sample than in the 2C *Coffea canephora*. The same trend was observed in silverskin samples. *Coffea arabica* samples reached higher values of L*, a*, and b* compared to 2S *Coffea canephora* samples. Further comparison among coffee co-products showed that silverskin samples had a higher value of L* and b* than cascara samples. Gocmen et al. [3] state that adding silverskin to products, such as cookies, can cause a significant color change in the final product. Authors reported that the silverskin addition caused the darker color of bakery products than the addition of cocoa powder. Other studies with similar structures reported similar results [64,65].

### 3.9. Sensory Analysis

**Figure 2:**
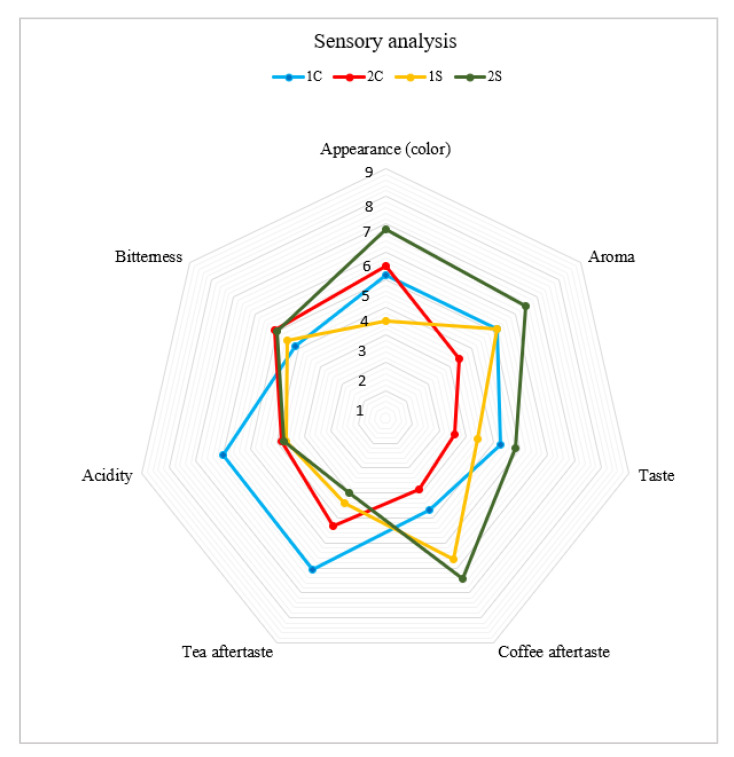
Results of sensory analysis of coffee co-products. Note: 1S—silverskin *Coffea arabica*; 2S—silverskin *Coffea canephora*; 1C—cascara *Coffea arabica*; 2C—cascara *Coffea canephora*.

According to sensory evaluation techniques, the analysis evaluated the following parameters: appearance (color), aroma, taste, coffee aftertaste, tea aftertaste, acidity, and bitterness [23,24,25,26,27,28]. The results of the sensory analysis are shown in Figure 2.

**Figure 3:**
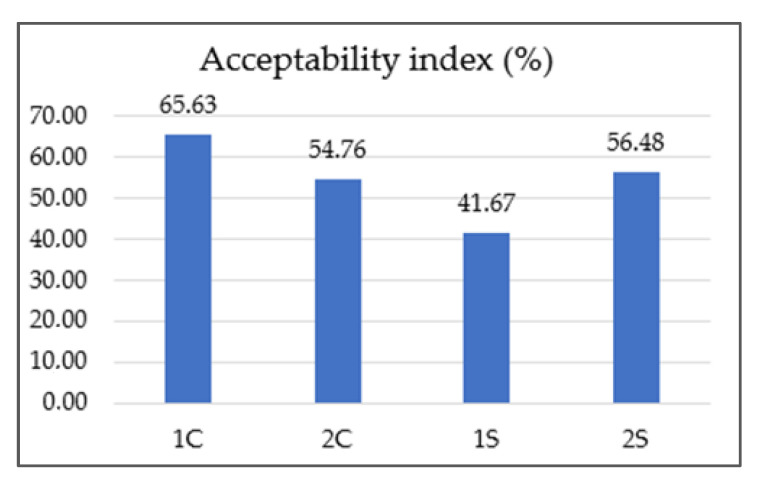
Results of acceptability index of coffee co-products extracts. Note: 1S—silverskin (thin layer) *Coffea arabica*; 2S—silverskin (thin layer) *Coffea canephora*; 1C—cascara (coffee husks) *Coffea arabica*; 2C—cascara (coffee husks) *Coffea canephora*.

Sample 2S (silverskin *Coffea canephora*) received the highest score for appearance (color), aroma, taste, and coffee aftertaste. Sample 1C (cascara *Coffea arabica*) received a higher score in tea aftertaste and acidity. Sample 2C (cascara *Coffea canephora*) received the best evaluation of bitterness based on a panel of evaluators. On the contrary, sample 1S received the lowest rating score for appearance (color), tea aftertaste, and bitterness. Sample 2C received the lowest taste rating score. DePaula et al. [66] and Sholichah et al. [45] report that the fermentation process and the specific conditions affect the flavor profile of cascara extract. ANOVA analysis—Duncan and REGWQ tests for Sensory analysis of coffee co-products are shown in the Supplementary Material (Table S2). The results of the acceptability index are represented in Figure 3.

**Figure 4:**
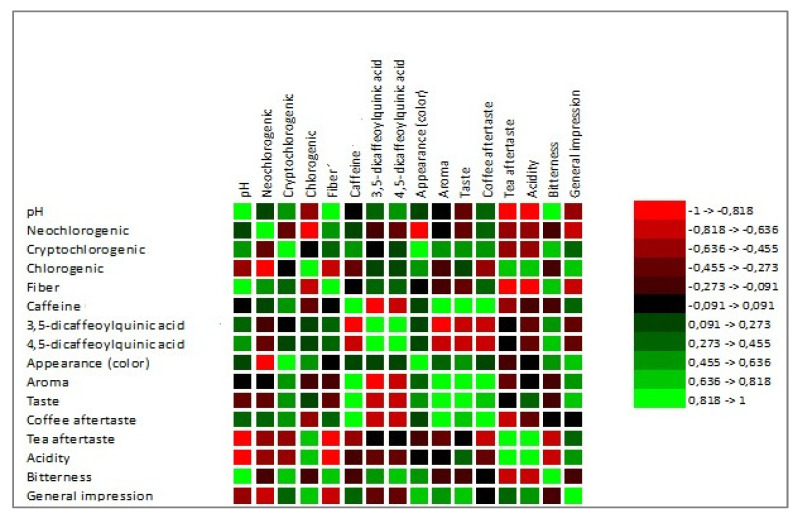
Visualization of Pearson’s correlation. Note: neochlorogenic, cryptochlorogenic, chlorogenic, caffeine, 3.5—dicaffeoylquinic acid, and 4.5—dicaffeoylquinic acid are expressed in mg·g^−1^.

According to Meilgaard et al. [27], a sample is considered “well-accepted” if it gets an overall impression (acceptability index) score of 70% or more. Based on this, none of our tested co-product extracts reached this threshold (1S 41.76%, 2S 56.48%, 1C 66.63%, 2C 54.76%). The lowest-rated sample was 1S (silverskin *Coffea arabica*). On the contrary, the overall highest-rated sample was 1C (cascara *Coffea arabica*). A similar finding regarding the acceptability of cascara extracts was also described in the research DePaula et al. [66]. Abduh et al. [67] stated that based on the results of organoleptic tests, evaluators preferred the cascara drink without any addition, like honey or sugar, due to its fresh and sour taste. Martinez-Saez et al. [68] conducted a sensory evaluation of silverskin extract and reported that only 10% of respondents found the taste of pure silverskin extract acceptable. However, 85% of the respondents would consume the drink in combination with another ingredient, such as milk, sugar, lemon, or honey. This study also described better acceptance of *Coffea arabica* silverskin than *Coffea canephora* silverskin. Increasing acceptance of the silverskin extract by adding concentrated pineapple juice to enhance flavor and aroma was reported by Neves et al. [69]. The correlation between the chemical parameters and sensory attributes is shown in Figure 4.

The sensory analysis is necessary to emphasize that results depict the subjective opinions of evaluators. Pearson’s correlation showed certain relations between the chemical composition and sensory properties of cascara and silverskin. Pearson’s correlation showed a high positive correlation between pH and bitterness attribute (correlation coefficient 0.925). Regarding the pH parameter, we observed a correlation with cryptochlorogenic acid (0.624), 3.5-dicaffeoylquinic acid (0.450), and 4.5-dicaffeoylquinic acid (0.536). However, according to the correlation matrix, none of these three were statistically significant. The correlation test also revealed that panelists consider coffee co-products that contained more caffeine as more aromatic, which positively correlates with the general impression; however, not significantly (0.531).

Interestingly, evaluators did not report higher acidity with higher pH values. A similar fact was observed by Rao and Fuller [70] and Basheer et al. [71]. The authors did not report a positive correlation between pH and titrable acidity. 

Our research suggests that there might be several compounds that affect its sensory properties. To better understand these relationships, it is essential to further focus on chemical composition and sensory analysis, considering the botanical, geographical composition, and post-harvest processing.

## 4. Conclusions

This study focused on exploring the influence of the different botanical origins of silverskin and cascara on various parameters. Our results confirm significant differences between samples of various botanical origins in parameters such as water activity (a_w_), pH, and dry matter. The research has also shown that silverskin is rich in TAC (up to 58.17%). Cascara *Coffea canephora* showed the presence of polyphenols (49.135 g GAE·kg^−1^). Both coffee co-products are good sources of fiber (1S 64.725%, 2S 61.063%, 1C 37,573%, and 2C 63.753%). Silverskin had higher values of caffeine than cascara. Palmitic, stearic, oleic, linoleic, and arachidic acids were the most represented acids in the samples. For cascara samples, we can use claims “low fat” (1C 4.240 g·kg^−1^ and 2C 5.4 g·kg^−1^). Based on the sensory evaluation, no sample reached the acceptable index value of 70%. According to the sensory analysis, the best-rated sample was 1C, while 1S was the lowest-rated sample. Considering these findings from the pilot study, modifying the methodology of preparation extracts from coffee co-products to sensory evaluation for better consumption acceptability is necessary. Further research is needed with a trained sensory evaluation panel, as well as consumer testing to determine the acceptability of coffee by-product extracts and coffee by-product-based beverages is needed. The insights gained from this study may assist in authenticating the origin of coffee co-products and examining or establishing the suitability of the methods used in the post-harvest processing of coffee co-products. It can also provide essential data for the suitability of the technological processing of the given products when incorporating them into food. Additionally, incorporating coffee co-products will ensure environmental food sustainability by lowering waste disposal and improving the food’s functional and nutritional efficacy. The current data in this research highlight the importance of creating quality limits and standards focused on rating coffee co-products’ microbiological, chemical, and physical safety and providing high-quality final raw materials.

## Figures and Tables

**Table 1:** Detailed description of analyzed samples.

Parameter	1S	2S	1C	2C
Cultivar	*C. arabica*	*C. canephora*	*C. arabica*	*C. canephora*
Date of roast *, harvest **	2021 *	2023 *	2020 **	2020 **
Roasting *, post-harvest ** process	Medium *	Medium *	Natural/dry **	Natural/dry **
Geographical origin	Panama	India	Panama	India
Variety	mix	mix	Maragogype	Catmar
Altitude	-	-	1300 mamsl	600–850 mamsl

Note: 1S—Silverskin (thin layer) *Coffea arabica*; 2S—silverskin (thin layer) *Coffea canephora*; 1C—cascara (coffee husks) *Coffea arabica*; 2C—cascara (coffee husks) *Coffea canephora*, mamsl: meters above mean sea level; date of roast *; date of harvest **; roasting process *; post-harvest process **.

**Table 2:** Physical and chemical parameters in coffee co-products.

Parameter	1S	2S	1C	2C
a_w_	0.18 ± 0.01 ^a^	0.28 ± 0.01 ^b^	0.64 ± 0.04 ^d^	0.39 ± 0.01 ^c^
pH	4.62 ± 0.03 ^b^	5.90 ± 0.02 ^c^	4.22 ± 0.03 ^a^	5.97 ± 0.02 ^d^
Dry matter (%)	92.70 ± 0.11 ^c^	93.91 ± 0.14 ^d^	85.16 ± 0.12 ^a^	89.60 ± 0.01 ^b^
TAC (%)	58.17 ± 1.28 ^d^	46.65 ± 1.20 ^c^	36.54 ± 1.84 ^a^	41.12 ± 2.11 ^b^
TPC (g GAE. kg^−1^)	1.64 ± 0.18 ^a^	11.15 ± 0.69 ^b^	2.59 ± 0.17 ^a^	49.14 ± 1.35 ^c^
Fiber (%)	64.73 ± 0.19 ^d^	61.06 ± 0.04 ^b^	37.57 ± 0.37 ^a^	63.75 ± 0.04 ^c^
Neochlorogenic acid (mg·g^−^)	0.34 ± 0.01 ^c^	0.11 ± 0.01 ^b^	0.09 ± 0.01 ^a^	0.12 ± 0.01 ^b^
Cryptochlorogenic acid (mg·g^−1^)	0.21 ± 0.09 ^ab^	0.80 ± 0.09 ^c^	0.18 ± 0.07 ^a^	0.38 ± 0.09 ^b^
Chlorogenic acid (mg·g^−1^)	0.38 ± 0.09 ^a^	0.50 ± 0.09 ^ab^	0.60 ± 0.09 ^b^	0.52 ± 0.09 ^ab^
Caffeine (mg·g^−1^)	5.77 ± 0.09 ^c^	8.01 ± 0.09 ^d^	4.12 ± 0.09 ^b^	0.48 ± 0.09 ^a^
3,5-dicaffeoylquinic acid (mg·g^−1^)	n.d.	n.d.	n.d.	0.27 ± 0.02 ^d^
4,5-dicaffeoylquinic acid (mg·g^−1^)	n.d.	0.07 ± 0.02 ^b^	n.d.	0.03 ± 0.01 ^a^

Note: a, b, c, d = groups within a column with different superscripts differ significantly at *p* ≤ 0.05; 1S—Silverskin (Thin layer) *Coffea arabica*; 2S—Silverskin (Thin layer) *Coffea canephora*; 1C—Cascara (Coffee husks) *Coffea arabica*; 2C—Cascara (Coffee husks) *Coffea canephora,* n.d. not detectable. Duncan and REGWQ tests were used for data processing.

**Table 3:** Content of PUFA, MUFA, SFA, ratio Σn3/Σn6, and ratio Σn6/Σn3 in coffee co-products.

Sample ID	PUFA	MUFA	SFA	Ratio Σn3/Σn6	Ratio Σn6/Σn3
1S	26.81 ± 0.25 ^c^	5.11 ± 0.27 ^b^	67.53 ± 0.03 ^b^	0.08 ± 0.01 ^c^	12.29 ± 0.08 ^a^
2S	22.74 ± 0.03 ^a^	5.49 ± 0.17 ^b^	71.77 ± 0.14 ^a^	0.17 ± 0.01 ^b^	6.06 ± 0.05 ^b^
1C	51.91 ± 0.30 ^a^	5.55 ± 0.07 ^b^	42.54 ± 0.37 ^d^	0.67 ± 0.02 ^a^	1.50 ± 0.03 ^c^
2C	29.12 ± 0.12 ^b^	19.45 ± 0.79 ^a^	51.44 ± 0.66 ^c^	n.d.	n.d.

Note: a, b, c, d = groups within a column with different superscripts differ significantly at *p* ≤ 0.05; 1S—silverskin (thin layer) *Coffea arabica*; 2S—silverskin (thin layer) *Coffea canephora*; 1C—cascara (coffee husks) *Coffea arabica*; 2C—cascara (coffee husks) *Coffea canephora*; n.d. not detectable. Duncan and REGWQ tests were used for data processing.

**Table 4:** Results of instrumental color determination.

Sample	L* (D65)	a* (D65)	b* (D65)	C*	h˚
1S	43.10 ± 0.07 ^a^	9.48 ± 0.06 ^b^	25.91 ± 0.08 ^a^	27.58 ± 0.09 ^a^	69.91 ± 0.05 ^b^
2S	36.36 ± 0.05 ^b^	6.38 ± 0.03 ^c^	19.99 ± 0.07 ^c^	19.08 ± 0.07 ^d^	70.47 ± 0.02 ^a^
1C	30.07 ± 0.08 ^c^	10.44 ± 0.09 ^a^	19.29 ± 0.11 ^b^	21.93 ± 0.14 ^b^	61.58 ± 0.05 ^c^
2C	28.65 ± 0.11 ^d^	10.35 ± 0.01 ^a^	17.52 ± 0.06 ^d^	20.34 ± 0.06 ^c^	59.42 ± 0.09 ^d^

Note: a, b, c, d = groups within a column with different superscripts differ significantly at *p* ≤ 0.05; L* = measured lightness, a* = measured redness, b* = measured yellowness; C* = calculated chroma; h˚ = calculated hue angle; 1S—silverskin (thin layer) *Coffea arabica*; 2S—silverskin (thin layer) *Coffea canephora*; 1C—cascara (coffee husks) *Coffea arabica*; 2C—cascara (coffee husks) *Coffea canephora*. Duncan and REGWQ tests were used for data processing.

## Data Availability

Not applicable.

## Supplementary Materials

The following supporting information can be downloaded at: https://www.mdpi.com/article/10.3390/foods12142675/s1, Figure S1: Picture of analyzed samples (cascara—coffee husks), Figure S2: Picture of analyzed samples (silverskin—thin layer), Figure S3: Chromatograms of fatty acids measurements by GC-FID (cascara—coffee husks), Figure S4: Chromatograms of fatty acids measurements by GC-FID (silverskin—thin layer), Table S1: Profile of fatty acids determined by GC-FID, Table S2: Sensory analyses of coffee co-products.

## Abbreviations

1C Cascara (coffee husks) *Coffea arabica*; 1S Silverskin (thin layer) *Coffea arabica*; 2C Cascara (coffee husks) *Coffea canephora*; 2S Silverskin (thin layer) *Coffea canephora*; aw water activity; DM dry matter; EFSA European Food Safety Authority; FAME Fatty Acid Methyl Esters Profile; GC-FID Gas Chromatography With Flame Ionization Detection; HPLC-DAD (High-Performance Liquid Chromatography with Diode-Array Detection); mamsl meters above mean sea level; MUFA monounsaturated fatty acids; n.d. not detectable; PUFA polyun saturated fatty acids; REGWQ The Ryan, Einot, Gabriel, Welsh, Studentized Range Q test; SFA saturated fatty acid; TAC total antioxidant capacity; TPC total polyphenols content.
