# Supplementary material for: Quality Attributes and Sensory Acceptance of Different Botanical Coffee Co-Products
Source: Foods. 2023 Jul 11;12(14):2675. doi: 10.3390/foods12142675 (PMC10378423; doi:10.3390/foods12142675)
Supplement: Supplementary file 1 [file foods-12-02675-s001.zip › foods-2401977-supplementary.pdf]

RMIT Classification: Trusted

Figure S1 Picture of analyzed samples (cascara- coffee husks)

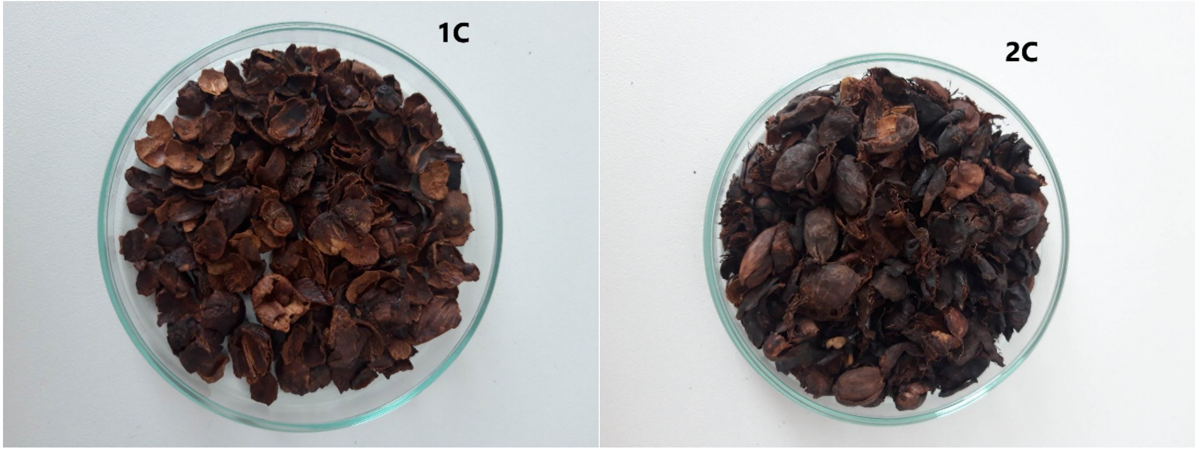

Note: 1C cascara *Coffea arabica*; 2C cascara *Coffea canephora*

Figure S2 Picture of analyzed samples (silverskin- thin layer)

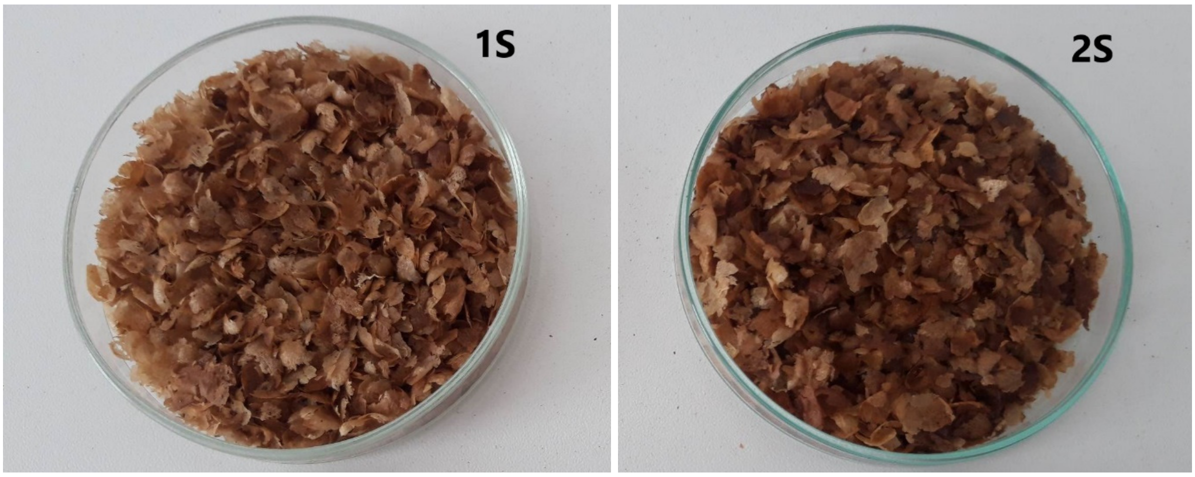

Note: 1S silverskin *Coffea arabica*; 1S silverskin *Coffea canephora*

RMIT Classification: Trusted

Figure S3 Chromatograms of fatty acids measurements by GC-FID (cascara- coffee husks)

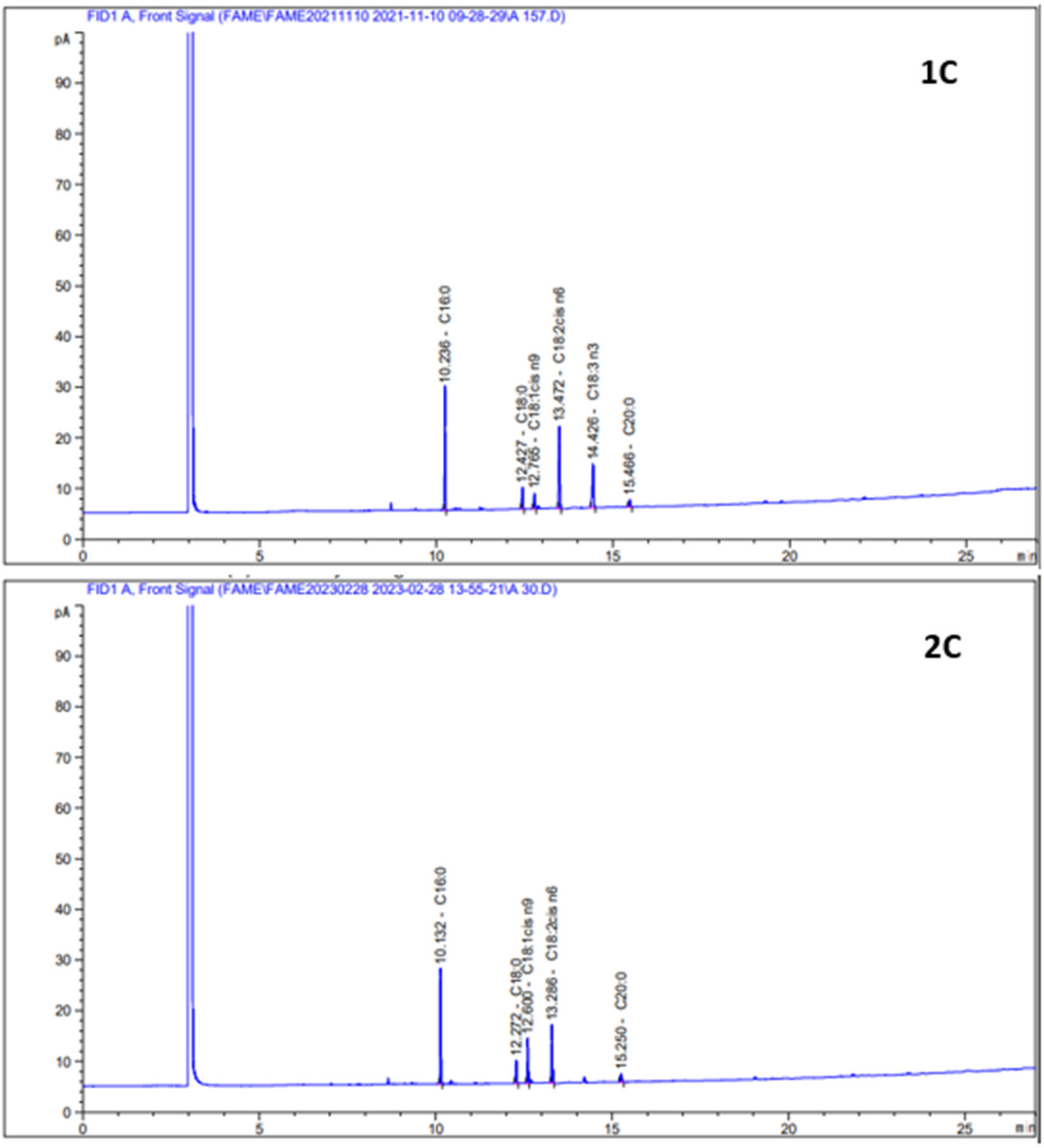

Note: 1C cascara *Coffea arabica*; 2C cascara *Coffea canephora*

Figure S4 Chromatograms of fatty acids measurements by GC-FID (silverskin- thin layer)

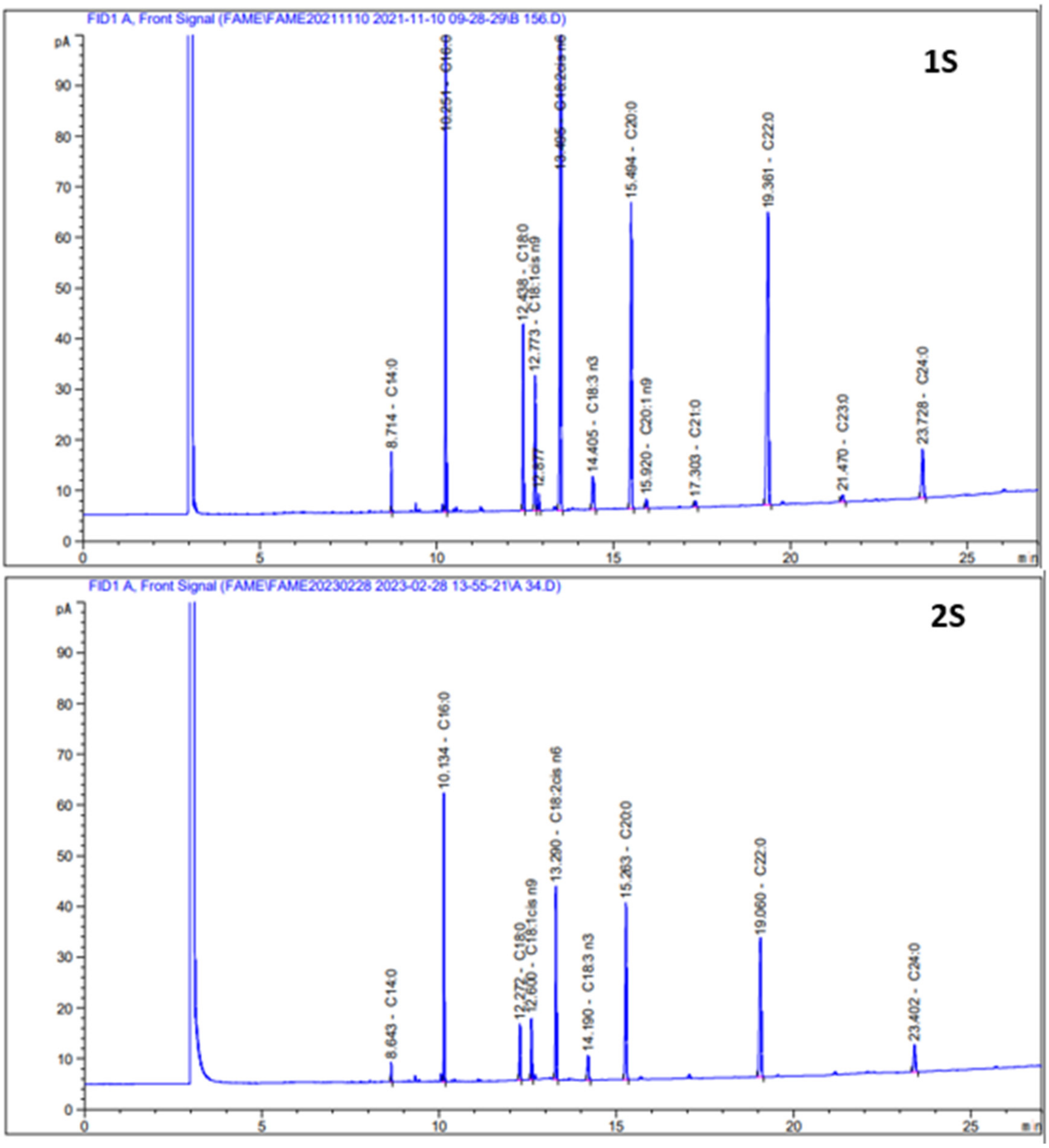

Note: 1S silverskin *Coffea arabica*; 2S silverskin *Coffea canephora*

Table S1 Profile of fatty acids determined by GC-FID.

**Fatty acids (%)**	**Samples**			
	1S	2S	1C	2C
butyric acid	0.000	0.000	0.000	0.000
caproic acid	0.000	0.000	0.000	0.000
caprylic acid	0.000	0.000	0.000	0.000
capric acid	0.000	0.000	0.000	0.000
undecanoic acid	0.000	0.000	0.000	0.000
lauric acid	0.000	0.000	0.000	0.000
tridecanoic acid	0.000	0.000	0.000	0.000
myristic acid	1.033	0.942	0.000	0.000
myristoleic acid	0.000	0.000	0.000	0.000
pentadecanoic acid	0.000	0.000	0.000	0.000
cis-10-pentadecenoic acid	0.000	0.000	0.000	0.000
palmitic acid	25.434	18.353	31.533	36.195
palmitoleic acid	0.000	0.000	0.000	0.000
heptadecanoic acid	0.000	0.000	0.000	0.000
cis-10-heptadecenoic acid	0.000	0.000	0.000	0.000
stearic acid	6.146	4.889	7.724	10.322
elaidic acid	0.000	0.000	0.000	0.000
oleic acid	4.706	5.494	5.553	19.449
unidentified	0.553	0.000	0.000	0.000
linolelaidic acid	0.000	0.000	0.000	0.000
unidentified	0.000	0.000	0.000	0.000
linoleic acid	24.788	19.518	31.181	29.116
γ-linolenic acid	0.000	0.000	0.000	0.000
α-linolenic acid	2.018	3.222	20.729	0.000
arachidic acid	13.701	21.572	3.281	4.919
cis-11-eicosenoic acid	0.402	0.000	0.000	0.000
cis-11,14-eicosadienoic acid	0.000	0.000	0.000	0.000
heneicosanoic acid	0.280	0.000	0.000	0.000
cis-8,11,14-eicosatrienoic acid	0.000	0.000	0.000	0.000
arachidonic acid	0.000	0.000	0.000	0.000
cis-11,14,17-eicosatrienoic acid	0.000	0.000	0.000	0.000
cis-5,8,11,14,17-eicosapentaenoic acid (EPA)	0.000	0.000	0.000	0.000
behenic acid	17.463	21.294	0.000	0.000
erucic acid	0.000	0.000	0.000	0.000
cis-13,16-docosadienoic acid	0.000	0.000	0.000	0.000
tricosanoic acid	0.413	0.000	0.000	0.000
unidentified	0.000	0.000	0.000	0.000
unidentified	0.000	0.000	0.000	0.000
lignoceric acid	3.064	4.717	0.000	0.000
unidentified	0.000	0.000	0.000	0.000
nervonic acid	0.000	0.000	0.000	0.000
cis-4,7,10,13,16,19-docosahexaenoic acid (DHA)	0.000	0.000	0.000	0.000

Note: 1S silverskin *Coffea arabica*; 2S silverskin *Coffea canephora*,1C cascara *Coffea arabica*; 2C cascara *Coffea canephora*

Table S2 Sensory analyses of coffee co-products

Category	Appearance (color)	Aroma	Taste	Coffee after taste	Tea after taste	Acidity	Bitterness	General impression
1S	3.500 ^{a}	5.167 ^{ab}	3.417 ^{a}	5.667 ^{b}	3.417 ^{a}	3.667 ^{a}	4.500 ^{a}	3.333 ^{a}
2S	6.833 ^{b}	6.500 ^{b}	4.833 ^{a}	6.417 ^{b}	3.000 ^{a}	3.750 ^{a}	5.000 ^{a}	5.083 ^{a}
1C	5.167 ^{ab}	5.167 ^{ab}	4.250 ^{a}	3.667 ^{a}	6.083 ^{b}	6.000 ^{b}	4.167 ^{a}	5.250 ^{a}
2C	5.500 ^{ab}	3.417 ^{a}	2.583 ^{a}	2.833 ^{a}	4.333 ^{a}	3.833 ^{a}	5.083 ^{a}	3.833 ^{a}

Note: a, b = groups within a column with different superscripts differ significantly at $p \leq 0.05$; 1S silverskin *Coffea arabica*; 2S silverskin *Coffea canephora,*1C cascara *Coffea arabica*; 2C cascara *Coffea canephora*
